# Author’s Reply to “Methodological considerations in evaluating socioeconomic outcomes after atrial fibrillation ablation”

**DOI:** 10.1016/j.hroo.2026.07.020

**Published:** 2026-07-23

**Authors:** Youlin Koh, Michael Wong

**Affiliations:** 1 Department of Cardiology, Royal Melbourne Hospital, Parkville, Victoria, Australia; 2 Department of Medicine, University of Melbourne, Victoria, Australia; 3 Department of Cardiology, Western Health, Melbourne, Victoria, Australia

We write to thank authors Riaz and Saindad for their feedback piece “Methodological considerations in evaluating socioeconomic outcomes after atrial fibrillation ablation.”^1^

First, we acknowledge that a population derived from randomized controlled trials inherently includes selection bias against less motivated or engaged individuals. However, we have aimed to mitigate this by including a population managed in the public health system, many of whom are initiated into the system by presenting in acute situations with their atrial fibrillation, and are therefore, a more symptomatic cohort. For our local health system, this has drawn attention to the need to improve access to atrial fibrillation catheter ablation in the public system so that we may better “capture” less socioeconomically advantaged individuals.

Second, we agree that there is a longstanding gap in equal sex representation in cardiac electrophysiology and also cardiology studies in general. This may be partially because of the higher incidence and prevalence of atrial fibrillation in men;^2^ a study recruiting by disease status is more likely to recruit male participants. We also recognize that to counter this, future studies should preferentially recruit women to achieve equal representation. We have been fortunate to collaborate with colleagues who are actively pursuing this in randomized controlled trial design.^3^

Third, we have chosen to combine 2 populations using different quality-of-life measures to increase the statistical power of our sample. We have also chosen to focus our presentation on the change in score rather than absolute values, to obtain a bigger-picture relative change owing to differences in boundary values between the 2 instruments. We also acknowledge that this is an imperfect metric, and future studies undertaken by our author group will aim to standardize quality-of-life measurements used between cohorts to increase replicability and comparison over time.

We are grateful to have received this constructive feedback on how to further improve investigations into socio-economic status and disease outcomes, which are often difficult to perform consistently owing to the heterogeneity of measured factors.
